# Reducing progression of experimental lupus nephritis via inhibition of the B7/CD28 signaling pathway

**DOI:** 10.3892/mmr.2015.3953

**Published:** 2015-06-18

**Authors:** LI HUANG, YONG KONG, JING WANG, JIE SUN, QIN SHI, YU-HUA QIU

**Affiliations:** 1 Department of Internal Medicine, Children's Hospital of Soochow University, Suzhou, Jiangsu 215000, P.R. China; 2 Department of Immunology, Medical College, Soochow University, Suzhou, Jiangsu 215123; 3 Laboratory Animal Center of Soochow University, Suzhou, Jiangsu 215123, P.R. China; 4 Division of Nano Biomedicine, Suzhou Institute of Nano-Tech and Nano-Bionics, Chinese Academy of Sciences, Suzhou, Jiangsu 215000, P.R. China; 5 Department of Orthopedics, First Affiliated Hospital of Soochow University, Suzhou, Jiangsu 215123, P.R. China

**Keywords:** lentivirus, B7-1, RNA interference, systemic lupus erythematosus, co-stimulatory molecules

## Abstract

The aim of the present study was to evaluate the effects of the B7/cluster of differentiation (CD)28 signaling pathway on experimental lupus nephritis and examine the molecular mechanism involved by inhibiting the B7/CD28 signaling pathway. A lupus nephritis model in C57BL/6 J mice was induced via intraperitoneal injection of pristane. A recombinant B7-1 short hairpin RNA (shRNA) lentivirus vector was constructed by synthesis and splicing. A neutralizing mouse anti-human B7-1 antibody termed 4E5 was also prepared. The mouse model of lupus nephritis was treated with B7-1 shRNA and 4E5 via injection through the tail vein. The silencing effects of B7-1 shRNA lentiviral infection on target molecules were evaluated using immunofluorescence and flow cytometry. The levels of protein in the urine were detected using Albustix test paper each month over 10 months. The concentration of interleukin (IL)-4 and interferon-γ in the serum was determined using an ELISA. The immune complex (IC) deposits in the kidney were analyzed using direct immunofluorescence. The results demonstrated that the C57BL/6 J mouse lupus nephritis model was successfully constructed with immune cells activated in the spleen of the mice, increases in the concentration of anti-nuclear antibody (ANA) and anti-double stranded DNA antibodies as well as positive IC formation. Following B7-1 shRNA lentivirus or 4E5 treatment, CD11b^+^B7-1^+^, CD11c^+^B7-1^+^ and CD21^+^B7-1^+^ cells in the spleen of the mice were significantly reduced. The concentration of ANA and IL-4 in the serum was also decreased. The concentration of urine protein was reduced and it was at its lowest level in the 4E5 early intervention group. It was also revealed that the immunofluorescence intensity of the IC deposits was weak in the 4E5 early intervention group. In conclusion, inhibiting the B7-1/CD28 signaling pathway is able to alleviate experimental lupus nephritis and provides an experimental basis for the therapeutic use of blocking the B7-1/CD28 signaling pathway in human lupus nephritis and other autoimmune disorders.

## Introduction

Systemic lupus erythematosus (SLE) is a complex autoimmune disease, the origin of which remains to be elucidated. It is considered to affect virtually every organ in the human body (1). SLE is primarily caused by autoantibodies and immune complex (IC) deposition. Enhanced apoptosis in conjunction with the defective clearance of apoptotic cells results in high levels of autoantibodies (2). Lupus nephritis (LN) is a major cause of morbidity and mortality in patients with SLE (3). The general consensus is that 60% of patients with lupus may develop clinically relevant nephritis at a certain time-point during the course of their illness (4). The prompt recognition and treatment of renal disease is important, as an early response to therapy is correlated with an improved outcome (5). Similar to other inflammatory and autoimmune conditions, the pathogenic processes which contribute to SLE involve complex interactions between immunocytes and nonimmunocytes, including endothelial and epithelial cells, through a number of receptor-ligand systems (1).

Signal transduction through CD28 is important in regulating the initial response of a T cell to an antigen (6). To date, two distinct CD28 ligands, B7-1 (CD80) and B7-2 (CD86), have been identified (7). These ligands also bind to cytotoxic T-lymphocyte-associated protein 4 (CTLA-4), a receptor closely associated with CD28, which is expressed on activated T cells (7). B7-1 is expressed on the surface of antigen-presenting cells (APCs), and its combination to the receptor CD28 is able to generate co-stimulatory signals, which are essential in the primary immune response (8). Without the co-stimulatory signal, T cells enter into a state of anergy, tolerance and even apoptosis (9). By contrast, the hyper-reaction of the B7/CD28 signal is closely associated with the occurrence of autoimmune diseases (10).

RNA interference (RNAi) is a process that is mediated by double stranded (ds)RNA dependent on small interfering RNA (siRNA) of 21–23 nucleotides, which is able to silence the gene expression of specific genes. It is also termed post-transcriptional gene silencing, as it is able to suppress target gene expression quickly, specifically and efficiently (11). It is well-established that specific antibody binding to an antigen is able to inhibit or promote various biological effects (12). Neutralization of B7 with anti-B7 antibody inhibits or downregulates its binding to its receptor CD28 (13) and therefore, delays the activation and immune response of B and T cells.

The present study was designed to determine the putative effect of preventative therapy with B7-1 short hairpin RNA (shRNA) or neutralizing anti-B7 antibody on a murine model of LN and to examine the potential mechanisms through which this therapy may be acting to improve clinical outcomes. C57BL6 J mice were treated with either B7-1 shRNA or neutralizing anti-B7 antibody to examine the implications of these results in the understanding of LN and the possible roles for B7/CD28.

## Materials and methods

### Reagents and antibodies

Pristane was purchased from Sigma-Aldrich (St. Louis, MO, USA). Phycoerythrin (PE)-conjugated monoclonal anti-mouse CD11b (cat. no. 12-0112), CD11c (cat. no. 12-0114), Myeloid differentiation antigen, Ly-6G (Gr1; cat. no. 12-5931) and CD21 (cat. no. 12-0212) antibody, allophycocyanin (APC)-conjugated monoclonal anti-mouse major histocompatibility complex (MHC)-II IgG (cat. no. 17-5321), fluorescein isothiocyanate (FITC)-conjugated monoclonal anti-mouse B7-1 and CD86 IgG (cat. nos. 11-0801 and 11-0862) and mouse interleukin (IL)-4 and interferon (IFN)-γ ELISA ready-set-go kit were purchased from eBioscience (San Diego, CA, USA), FITC-conjugated goat anti mouse immunoglobulin G (IgG) antibody were obtained from Jackson ImmunoResearch Labs (West Grove, Pennsylvania, USA).

### Cell culture

293T, L929 and K-562 cell lines (American Type Culture Collection, Manassas, VA, USA) were grown in RPMI-1640 (Life Technologies, Thermo Fisher Scientific, Grand Island, NY, USA) culture medium containing 10% fetal bovine serum (FBS; HyClone, Logan, UT, USA), 100 *μ*g/ml streptomycin and 100 U/ml penicillin (Sigma-Aldrich). The cells were maintained at 37°C with 5% CO_2_ and were progressively passaged every two days.

### Animals

All procedures were approved by the Guideline for the Care and Use of Laboratory Animals on the Chinese Medical Academy and the Soochow University Animal Care Committee (Suzhou, China). Specific pathogen-free C57BL/6 J female mice (7–8 weeks old; weighing 20–25 g) were obtained from Shanghai Laboratory Animal Centre (Shanghai, China) and were maintained in our animal facility under specific pathogen-free conditions. A 12-h day/night cycle was maintained during the entire course of the study at 18–22° and 50–60% humidity. Animals were housed in groups of five, fed a standard diet and had *ad libitum* access to water.

### Mouse anti-human B7-1 monoclonal antibody (mAb) production

The hybridoma cell line from our laboratory (14), which had been observed to secrete anti-human B7-1 antibody with the highest titers was mass cultured for hybridoma injection at the 5th-6th passage. A total of 20 BALB/c mice (female, 6–8 weeks old) were intraperitoneally injected with sterile paraffin oil (0.5 ml per mouse) seven days prior to hybridoma injection. Each mouse was injected with 1–2×10^6^ hybridoma cells. After 7–10 days, ascites were collected and centrifuged at 10,000 × g for 30 min to obtain the supernatant. The supernatant of the ascites was further purified with a protein G sepharose 4B column using a fast protein liquid chromatography system (GE Pharmacia, Ramsey, MN, USA) according to the manufacturer's instructions. The purity and concentration of the purified mAb was analyzed using 10% SDS-PAGE (Bio-Rad Laboratories, Inc., Hercules, CA, USA) and the Bradford protein assay (15), respectively.

### Lentivirus-mediated shRNA knockdown of B7-1 gene expression

The recombinant lentivirus was constructed according to the manufacturer's instructions with certain modifications. Briefly, four pairs of shRNA fragments were hybridized with synthesized sense and anti-sense oligonucleotides (Table I). This hybridized B7-1 shRNA fragment was cloned into the pGLV-green fluorescent protein (GFP) plasmid. 293T cells were co-transfected with 4 *μg* pGLV-GFP, *4 μg* pLV/Helper-SL3, 4 *μg* pLV/Helper-SL4 and 4 *μg* pLV/Helper-SL5 along with 40 *μ*l Lipofectamine 2000 (Invitrogen Life Technologies, Carlsbad, CA, USA). This recombinant lentivirus was designated B7-1 lenti. The titers of B7-1 lenti were determined with a GFP assay. Negative control lenti contained a scrambled shRNA fragment, an irrelevant shRNA with random nucleotides with a GC ratio similar to that of the B7-1 shRNA. When the L929 cell line reached 60–70% confluence, lentiviral vectors were added to the culture (serum-free medium) at a multiplicity of infection of 20, 40 and 60 in the presence of 5 ng/ml polybrene. Half of the medium was replaced every other day and the cultures were examined by fluorescent microscopy (IX71; Olympus, Tokyo, Japan) two days following transfection.

### Establishment of pristane-induced lupus model

C57BL/6 J mice were inoculated once with 0.5 ml pristane (2,6,10,14-tetramethyl-pentadecane; Sigma-Aldrich). The mice were divided into four groups: B7-1 antibody, IgG isotype, B7-1 shRNA and wild-type group. The B7-1 antibody mice were treated with 200 *μg* B7-1 antibody through an intravenous injection in the tail on day 1, 3, 5, 8 and 15 every month for three months after the pristane injection. The IgG isotype mice were treated with IgG control in parallel to the B7-1 antibody mice. The B7-1 shRNA mice were treated with 0.4×10^8^ TU LV-B7-1 shRNA on days 1 and 60 through an intravenous injection in the tail following pristane injection. The mice were bled every month following pristane inoculation and the sera were frozen for analysis of the autoantibodies. All mice were monitored for proteinuria once a month and were sacrificed via cervical dislocation at 8 or 10 months to harvest their kidneys. Kidney disease was assessed in mice treated with B7-1 antibody, B7-1 shRNA and in wild-type littermates. Proteinuria was measured on a 0–4 scale using a colorimetric assay strip for albumin (Albustix; Bayer, Elkhart, IN, USA), with scoring performed as follows: 0, (absent); 1, 30 mg/dl (mild); 2, 100 mg/dl (moderate); 3, 300 mg/dl (moderate to severe); and 4, 2000 mg/dl (severe). The experiment included 60 mice, which were allocated into six groups (10 mice per group).

### Phenotypic spleen population analysis by flow cytometry

For the collection of spleen cells, tissue of the spleen was gently scraped off with scissors. Cell suspensions were then passed through a nylon filter with 100-*μ*m pore size (Merck Millipore, Darmstadt, Germany). The spleen cells were pelleted by centrifugation at 1,000 × g for 5 min and then re-suspended in ammonium chloride-potassium lysis buffer (Amersham Pharmacia Biotech, Piscataway, NJ, USA) to remove erythrocytes. Following centrifugation for 10 min at 1,200 × g, the spleen cells were washed twice with RPMI-1640 medium with 5% FBS. The cells were co-stained with PE-conjugated monoclonal antimouse CD11b IgG and FITC-conjugated anti-B7-1 antibody, PE-conjugated anti-CD11c antibody and FITC-conjugated anti-B7-1 antibody, or PE-conjugated anti-CD21 antibody and FITC-conjugated anti-B7-1 antibody, respectively. In other experiments, the spleen cells were stained with PE-conjugated anti-CD11b antibody, PE-conjugated anti-CD11c antibody, PE-conjugated anti-CD21 antibody, PE-conjugated anti-Gr1 antibody, respectively, or co-stained with PE-conjugated anti-CD21 antibody and FITC-conjugated anti-CD86 antibody, or PE-conjugated anti-CD21 antibody and FITC-conjugated anti-APC-MHC-II antibody, respectively. Stained cells were analyzed using a BD FACSCalibur flow cytometer (BD Biosciences, Mountain View, CA, USA) and CellQuest software version 1.0 (BD Biosciences).

### Renal histology

Paraffin sections (4-*μ*m thick) of kidneys fixed in 4% paraformaldehyde were stained with hematoxylin and eosin at room temperature for 10 min and 5 min, respectively (Sigma-Aldrich). The stained sections were scored for the following features on a 0–3 scale in a blinded manner: i) Glomerular activity score (GAS), which included glomerular proliferation, karyorrhexis, fibrinoid necrosis, cellular crescents, inflammatory cells and hyaline deposits; ii) tubulointerstitial activity score (TIAS), which included interstitial inflammation, tubular cell pyknosis, nuclear activation, cell necrosis and cell flattening, and epithelial cells or macrophages in tubular lumens; iii) chronic lesions score (CLS), which included glomerular scars, glomerulosclerosis, fibrous crescents, tubular atrophy, and interstitial fibrosis and iv) vascular lesion score, which included arterial/arteriolar lesions. The raw scores assigned by various readers were averaged to obtain a mean score for each of the individual features. The mean scores for individual features were calculated to obtain the four main scores (GAS, TIAS, CLS and vascular lesion score) and then all four scores were summed to determine the composite kidney biopsy score.

### Renal immunostaining

Frozen kidney sections were fixed with methanol and acetone (1:1) for 5 min. The slides were then washed and incubated with FITC-conjugated goat anti-mouse IgG (Sigma-Aldrich). The slides were assessed by three individuals in a blinded manner.

### Detection of autoantibodies against anti-nuclear antibody (A NA) and double stranded (dsDNA)

ANA and dsDNA levels were detected by indirect immunofluorescence according to the manufacturer's instructions (Euroimmun, Lübeck, Germany). Undiluted samples were layered on ANA slides and incubated for 30 min. Following washing, fluorescein-labeled anti-mouse globulin was added to each slide and incubated for a further 30 min. The slides were then embedded with Floursave mounting reagent (Merck Millipore) and examined under a fluorescence microscope (Olympus). Fluorescence intensity was scored as follows: 4+, (very bright green), 3+, (bright green), 2+, (green) and 1+, (faint green).

### Detection of cytokines IL-4 and IFN-γ in sera

Mice sera were collected eight months following pristane injection. A standard sandwich ELISA was used to measure cytokines IL-4 and IFN-γ in the sera. IL-4 and IFN-γ levels were measured using the mouse cytokine ready-set-go kit according to the manufacturer's instructions.

### Scanning electron microscopy (SEM)

The renal specimens were collected and fixed in 2.5% glutaraldehyde buffer (pH 7.4) for 2 h The renal specimens were washed twice with phosphate buffered saline (Bio-Rad Laboratories, Inc.) for 15 min and then dehydrated using serial dilutions of alcohol (50, 70, 80, 90, 95, 99 and 100%). Critical point drying of specimens was performed with liquid CO_2_ and specimens were sputter-coated with gold and examined using SEM (S-450; Hitachi Ltd., Tokyo, Japan).

### Statistical analysis

All results are expressed as the mean ± standard deviation. Levels of antibodies and cytokines, lymphocyte percentages and quantities, as well as renal scores were compared using Student's t-test or the Mann-Whitney U test. Frequencies of antibodies and proteinuria were compared using the two-sided Fisher's exact test. Statistical calculations were performed with Prism 5 software (GraphPad Software, La Jolla, CA, USA). P<0.05 was considered to indicate a statistically significant difference.

## Results

### B7-1 deficiency reduces proteinuria production

To determine whether the B7-1/CD28 signaling pathway is involved in the development of pristane-induced lupus, the B7-1/CD28 pathway was blocked in mice by treatment with neutralizing anti-mouse B7-1 antibody or B7-1 shRNA. All mice were bled prior to and at four and eight months post-inoculation of pristane and monitored for proteinuria. Proteinuria developed late and was alleviated in the pristane-inoculated B7-1 antibody or B7-1 shRNA mice as compared with that in the control littermates (Table II). At eight months post-inoculation, 50% of the mice in the control group, but none in the B7-1 antibody or B7-1 shRNA group, exhibited 100 mg/dl (moderate to severe) proteinuria (P<0.05). The frequency of moderate to severe proteinuria remained high in control mice at 10 months (P<0.005). None of the B7-1 antibody or B7-1 shRNA mice, but 63% of the control mice developed severe (300 mg/dl) proteinuria at 10 months post-inoculation (P<0.05).

### B7-1 deficiency reduces pristane-induced autoantibody production

Pristane-inoculated BALB/c mice have been observed to develop autoantibodies to several cellular antigens (Ags) (16,17). These antibodies were detected with an immunoprecipitation assay using a cell extract from the K-562 erythroleukemia cell line as a source of autoantigens. Generally, reactivity to cellular Ags was lower in the sera from pristane-inoculated B7-1 antibody mice and B7-1 shRNA mice than that in the sera from pristane-inoculated control mice (Table III). Serum IgG anti-dsDNA antibody levels, as measured by ELISA, were also lower in the B7-1 antibody mice and B7-1 shRNA mice those in the control mice at six months post-inoculation (P<0.05; Table IV).

### B7-1 deficiency alleviates IC deposits

**Figure 1:**
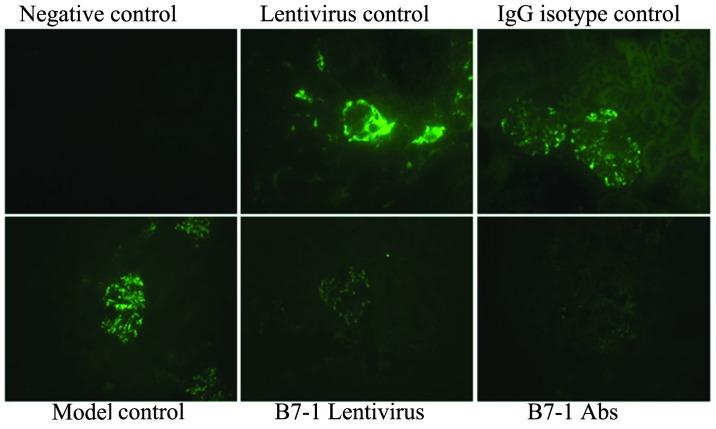
B7-1 deficiency alleviates IC deposits. After eight months of model induction, kidneys were collected from mice of five groups. Frozen kidney sections were fixed with methanol and acetone. Slides were washed and incubated with fluorescein isothiocyanate-conjugated goat anti-mouse IgG. Immunofluorescence revealed that IC deposits in renal glomeruli of the mice administered with B7-1 antibody or B7-1 short hairpin RNA were reduced compared with those in the model control mice (magnification, ×600). Abs, antibodies; IgG, immunoglobulin G; IC, immune complex.

As shown in Fig. 1, immunofluorescence revealed that IC deposits in the renal glomeruli of the mice administered with B7-1 antibody or B7-1 shRNA were fewer than in those of the control mice.

### Analysis of renal ultrastructure

**Figure 2:**
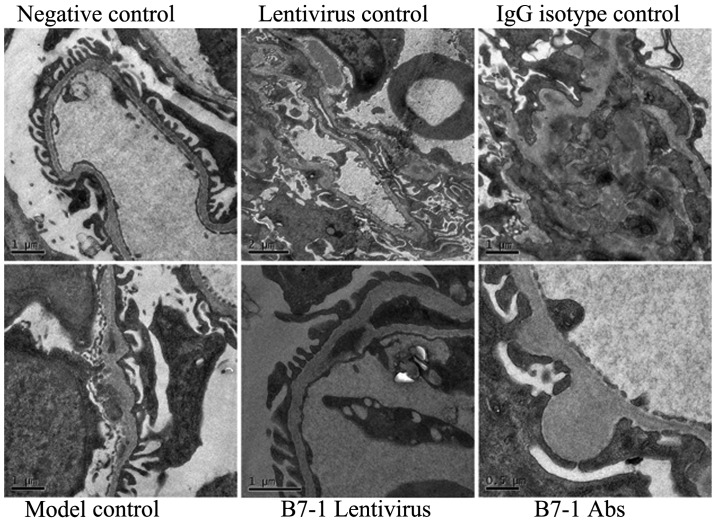
Analysis of renal ultrastructure using scanning electron microscopy. Renal specimens were collected and fixed in 2.5% glutaraldehyde buffer and then dehydrated by increasing the concentration of alcohol. Critical point drying of specimens was undertaken with liquid CO_2_ and specimens were sputter-coated with gold. In B7-1 lentivirus and B7-1 antibody intervention groups, there were less electron-dense deposits in glomerular endothelial cells compared with those in the model control group. Abs, antibodies; IgG, immunoglobulin G.

SEM showed electron-dense deposits in mouse glomerular endothelial cells in all model groups, and electron-dense deposits also formed in certain basement membranes (Fig. 2). Fused podocytes were observed in numerous glomeruli and false chorion were formed on the surface of podocytes, combined with basement membrane thickening. However, electron-dense deposits in the mouse glomerular endothelial cells in B7-1 lentivirus and B7-1 antibody treatment groups were fewer than those in the model control group (Fig. 2).

### B7-1 deficiency alleviates renal lesions

**Figure 3:**
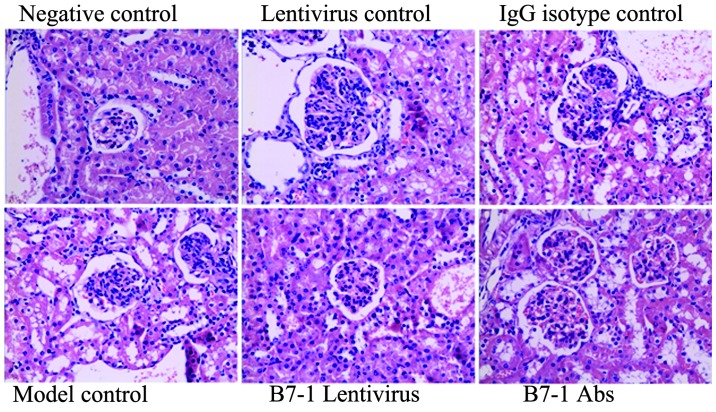
B7-1 deficiency alleviates renal lesions. Mice were sacrificed at 10 months to harvest kidneys and their renal histology was analyzed. Paraffin sections of kidneys fixed in 4% paraformaldehyde were stained with hematoxylin and eosin at room temperature for 10 min and 5 min, respectively. Representative photomicrographs (magnification, ×200) of renal histology for each group. Abs, antibodies; IgG, immunoglobulin G.

The mice were sacrificed at 10 months to harvest the kidneys and their renal histology was assessed (Fig. 3). Mild and focal mesangio-proliferative glomerulonephritis was observed in the majority of pristane-inoculated C57/BJ mice (Fig. 3) by light microscopy (CX41-12C02; Olympus); however, none of the B7-1 antibody or B7-1 shRNA mice had diffuse proliferative or chronic lesions. In the control group, however, >60% of the mice developed diffuse proliferative glomerulonephritis with fibrous crescents, glomerulosclerosis, tubular atrophy and interstitial fibrosis (Fig. 3). Another pristane-inoculated control mouse exhibited mild to moderate mesangio-proliferative lesions.

### Cytokine IL-4 and IFN-γ responses in B7-1-deficient mice

**Figure 4:**
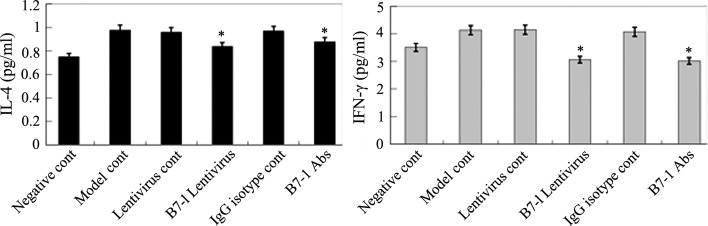
Cytokine IL-4 and IFN-γ responses in B7-1-deficient mice at 6 months following pristane inoculation. Quantitation of IL-4 and INF-γ expression in sera using ELISA. The experiment included 60 mice, which were divided into six groups (10 mice per group). *P<0.05 compared with the model control group. There was a significant reduction of INF-γ and IL-4 in the B7-1 lentivirus-treatment group (3.06±0.38 pg/ml and 0.84±0.052 pg/ml) and B7-1 antibody-treatment groups (3.02±0.55 pg/ml and 0.88±0.064 pg/ml) compared with those in the model control group (4.13±0.57 pg/ml and 0.98±0.035 pg/ml). IL, interleukin; IFN, interferon; Abs, antibodies; IgG, immunoglobulin G; cont, control.

Abnormalities in cytokine production contributes to the development of lupus (18). To determine whether the effects of B7-1/CD28 on the development of lupus are associated with abnormalities in cytokine production, the cytokine responses in the blood of pristane-inoculated B7-1 antibody, B7-1 shRNA and control mice were measured. As expected, IL-4 and IFN-γ levels were significantly decreased in the B7-1 antibody and B7-1 shRNA mice, as compared with those in the control mice. Such a cytokine profile, including increased type 1 cytokines, may contribute to the alleviation of autoimmune disease in B7-1 antibody and B7-1 shRNA mice, respectively (Fig. 4).

### Infiltration cell functions and quantities are reduced in B7-1 deficient mice

**Figure 5:**
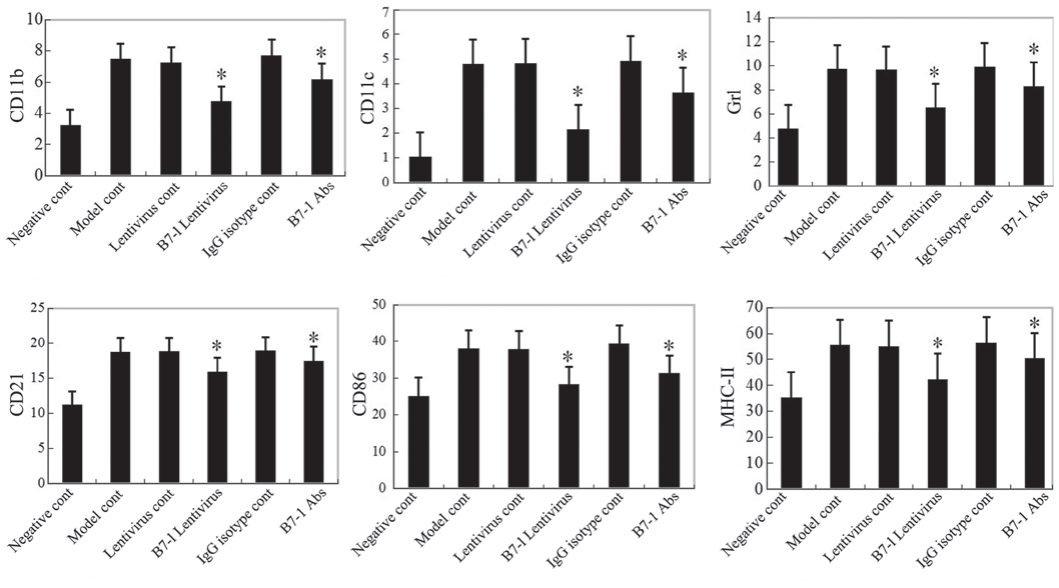
Infiltrating cell functions and numbers are reduced in B7-1-deficient mice six months following pristane inoculation. Expression of CD11b, CD11c, Gr1 and CD21 on cells in spleens from each group were detected using FACS. CD86 and MHC-II expression in CD21^+^ B cells from spleens of each group were also detected using FACS. *P<0.05 compared with the model control group, the expression of CD11b, CD11c, Gr1 and CD21 on cells in the spleen and the expression of CD86 and MHC-II on CD21^+^ B cells were all markedly inhibited in the B7-1 lentivirus-transfected group (473±0.48, 2.13±0.71, 6.50±1.58, 15.87±0.87, 28.21±1.32 and 42.26±3.17%) and B7-1 antibody treatment group (6.15±1.26, 3.65±0.88, 8.32±1.06, 1745±0.97, 31.21±2.35 and 50.26±3.25%). FACS, fluorescence-activated cell sorting; MHC, major histocompatibility complex; IgG, immunoglobulin G; Abs, antibodies; MHC, major histocompatibility complex; cont, control.

To assess whether the function of immune cells is also compromised in the B7-1 signaling pathway, spleen cells from control- or pristane-inoculated B7-1 antibody and B7-1 shRNA mice were analyzed for the activation and memory markers CD11b, CD11c, CD21, Gr1, CD86 and APC-MHC-II using flow cytometry (Fig. 5). Significant decreases in the induction of CD11b, CD11c, Gr1 and CD21 as well as CD86 and APC-MHC II on B cells were observed at six months post-inoculation in the B7-1 antibody and B7-1 shRNA treatment groups (P<0.05; Fig. 5), but no significant difference was observed at earlier time-points, including 12 h and 10–12 days post-inoculation (data not shown).

## Discussion

B7-1, which is expressed on the APC surface, is an important co-stimulatory molecule (19). Its receptor, CD28/CTLA-4 is expressed on T cells. The B7-1/CD28 signaling pathway is important in regulating the immune responses for the promotion of T-cell proliferation, T-helper (Th)1 and Th2 differentiation, antibody production and Ig type conversion, amongst others. However, excessive activation of the B7-1/CD28 signaling pathway may induce autoimmune diseases (20). Blocking this pathway may inhibit T- or B-cell responses, or even induce immune tolerance (21). The methods of inhibiting the B7/CD28 signaling pathway include the application of antisense oligonucleotides (19,22), gene knockout and monoclonal antibody blocking (23,24).

RNAi technology is currently widely used in molecular biology and cell biology. It is able to quickly and specifically inhibit the expression of target genes, therefore it has been used simply and effectively in gene knockout studies (25). In the present study, a murine B7-1 gene RNAi lentiviral vector was successfully constructed. It was able to effectively silence the L929 membrane-type molecule B7-1 to 73.2%. In addition, the functional mouse anti-human B7-1 monoclonal antibody, 4E5 was also prepared for comparison with the B7-1 shRNA. In the present study, the immunofluorescence and flow cytometry results revealed that in the B7-1 shRNA lentivirus infection g r o u p, C D11b ^+^B7-1^+^, CD11c^+^B7-1^+^ and CD21^+^B7-1^+^ cells in the spleen were significantly decreased compared with those in the control group. It was indicated that a recombinant lentivirus was able to effectively interfere with the APC surface expression of the B7-1 molecule.

Previous studies have revealed that T lymphocytes, in particular Th cells, are able to promote the proliferation and activation of B lymphocytes through the secretion of cytokines, resulting in a large number of pathogenic autoantibodies and ICs, leading to organ damage (26,27). Therefore, it is important in LN occurrence and development. In recent years, there have been a number of studies on the correlation between the balance of Thl/Th2 cells and SLE, or Thl/Th2 abnormal cytokine secretion and SLE. However, this correlation remains to be fully elucidated. Lazarus *et al* (28) and Hasegawa *et al* (29) observed that in humans and in a mouse model of lupus, when Th1 cell secretion of IFN-γ was increased, the development of the disease was more damaging, while Th2-cell secretion had the inverse function. Amel-Kashipaz *et al* (30) have indicated that the Th2-cell proportion in the peripheral blood of patients with SLE was significantly higher than that in the peripheral blood of normal control individuals. The authors suggested that Th2 dominance was correlated with the pathogenesis of SLE. The Th2 proportion skew may be due to the inhibition to Th1 proliferation and activation, Thl-type cytokine production or Thl cytokine gene transcription by cytokines secreted by Th2 (30,31). Szegedi *et al* (32) identified that Thl and Th2-type cytokine levels in the serum of patients with SLE were elevated and that Th1 and Th2 may function together in SLE. Consistent with the study by Szegedi *et al* (32), the present study revealed that in the model group, IL-4 and INF-γ levels in mouse serum were significantly higher than those in the normal control group. In the lentiviral interference group and B7-1 antibody early intervention group, IL-4 and INF-γ levels in mouse serum were significantly lower than those in the model group. Thus, it was suggested that mouse B7-1 shRNA lentivirus and a B7-1 antibody are able to inhibit the production of inflammatory cytokines, including IL-4 and INF-γ.

In the present study, it was identified that mouse B7-1 shRNA lentiviral vector intervention at the gene level or B7-1 monoclonal antibody intervention at the protein level were able to effectively inhibit the early activation of immune cells, reduce ANA antibody and proteinuria production and decrease IC deposition, thereby reducing renal pathological damage. However, the B7-1 monoclonal antibody was more effective than the B7-1 shRNA lentivirus. A possible reason for this difference may be that B7-1 antibodies directly bind and neutralize the antigen of B7-1, which is able to quickly react and affect inflammation and immune cell activation. However, the lentiviral vector interference effect was slower due to several indirect steps. Firstly, a delivery system is required to efficiently and safely transport siRNA for the target gene into the associated tissues or organs. Secondly, the effect of siRNA is generally considered to be sequence-specific; however, the effect is occasionally non-specific. Finally, the RNAi system may activate the IFN system and induce non-specific mRNA, which is not biologically efficient. With RNAi technology improving, such issues are likely to be resolved in the near future (33).

In conclusion, specific RNAi and antibodies are able to inhibit B7-1-mediated signaling pathways, thereby reducing the activation of immune cells and reversing pathological damage in a lupus nephritis model. At present, increasing numbers of treatments for SLE are under investigation (34); however, inhibition of co-stimulatory molecules is expected to present an efficacious, specific and non-toxic treatment for the biological intervention against SLE.

## Figures and Tables

**Table I:** Sequences of the four pairs of shRNA fragments used in the present study

shRNA	Sequence
shRNA-256	
Forward	5′-GATCCGCAATTGTCAGTTGATGCAGGTTCAAGAGACCTGCATCAACTGACAATTGCTTTTTTG-3′
Reverse	5′-AATTCAAAAAAGCAATTGTCAGTTGATGCAGGTCTCTTGAACCTGCATCAACTGACAATTGCG-3′
shRNA-409	
Forward	5′-GATCCGCCGTTACAACTCTCCTCATGTTCAAGAGACATGAGGAGAGTTGTAACGGCTTTTTTG-3′
Reverse	5′-AATTCAAAAAAGCCGTTACAACTCTCCTCATGTCTCTTGAACATGAGGAGAGTTGTAACGGCG-3′
shRNA-620	
Forward	5′-GATCCGGAAAGAGGAACGTATGAAGTTTCAAGAGAACTTCATACGTTCCTCTTTCCTTTTTTG-3′
Reverse	5′-AATTCAAAAAAGGAAAGAGGAACGTATGAAGTTCTCTTGAAACTTCATACGTTCCTCTTTCCG-3′
shRNA-807	
Forward	5′-GATCCGGCATCAATACGACAATTTCCTTCAAGAGAGGAAATTGTCGTATTGATGCCTTTTTTG-3′
Reverse	5′-AATTCAAAAAAGGCATCAATACGACAATTTCCTCTCTTGAAGGAAATTGTCGTATTGATGCCG-3′

shRNA, short hairpin RNA.

**Table II:** Comparison of the degree of proteinuria at 10 months post-inoculation (n=10).

Group	−	±	+	++	+++	++++
Negative control	7	2	1	0	0	0
Model control	0	0	0	1	5	3
Lentivrius control	0	0	0	3	4	2
B7-1 lentivrius^a^	0	2	1	5	2	0
IgG isotype control	0	0	0	3	3	3
B7-1 Abs^a,b^	0	3	5	2	0	0

a Compared with the model control group, P<0.05.

b Compared with the lentivirus intervention group and Abs delayed intervention group, P<0.05. Abs, antibodies; IgG, immunoglobulin G.

**Table III:** Titer of anti-nuclear antibody in the sera at six months post-pristane inoculation (n=10).

Group	−	+	++	+++
Negative control	9	1	0	0
Model control	0	1	1	7
Lentivrius control	0	2	2	5
B7-1 lentivrius^a^	1	4	4	1
IgG isotype control	0	1	3	5
B7-1 Abs^a,b^	3	6	1	0

a Compared with the model control group, P<0.05.

b Compared with the lentivirus intervention group and antibody delayed intervention group, P<0.05. Abs, antibodies; IgG, immunoglobulin G.

**Table IV:** Titer of anti-double-stranded DNA antibody in the sera at six months post-pristane inoculation (n=10).

Group	−	+	++	+++
Negative control	9	1	0	0
Model control	1	1	2	5
Lentivrius control	2	1	2	4
B7-1 lentivrius^a^	2	3	3	2
IgG isotype control	1	2	2	4
B7-1 Abs^a^	3	3	2	2

a Compared with the model control group, P>0.05. Abs, antibodies; IgG, immunoglobulin G.
